# Tracking-Based Interactive Assessment of Saccades, Pursuits, Visual Field, and Contrast Sensitivity in Children With Brain Injury

**DOI:** 10.3389/fnhum.2021.737409

**Published:** 2021-10-29

**Authors:** Scott W. J. Mooney, Nazia M. Alam, Glen T. Prusky

**Affiliations:** ^1^Burke Neurological Institute, White Plains, NY, United States; ^2^Blythedale Children’s Hospital, Valhalla, NY, United States; ^3^Weill Cornell Medicine, New York, NY, United States

**Keywords:** eye movements, contrast sensitivity, TBI—traumatic brain injury, cerebral visual impairment, brain injured children, measurement, psychophysics, visual field

## Abstract

Visual deficits in children that result from brain injury, including cerebral/cortical visual impairment (CVI), are difficult to assess through conventional methods due to their frequent co-occurrence with cognitive and communicative disabilities. Such impairments hence often go undiagnosed or are only determined through subjective evaluations of gaze-based reactions to different forms, colors, and movements, which limits any potential for remediation. Here, we describe a novel approach to grading visual health based on eye movements and evidence from gaze-based tracking behaviors. Our approach—the “Visual Ladder”—reduces reliance on the user’s ability to attend and communicate. The Visual Ladder produces metrics that quantify spontaneous saccades and pursuits, assess visual field responsiveness, and grade spatial visual function from tracking responses to moving stimuli. We used the Ladder to assess fourteen hospitalized children aged 3 to 18 years with a diverse range of visual impairments and causes of brain injury. Four children were excluded from analysis due to incompatibility with the eye tracker (e.g., due to severe strabismus). The remaining ten children—including five non-verbal children—were tested multiple times over periods ranging from 2 weeks to 9 months, and all produced interpretable outcomes on at least three of the five visual tasks. The results suggest that our assessment tasks are viable in non-communicative children, provided their eyes can be tracked, and hence are promising tools for use in a larger clinical study. We highlight and discuss informative outcomes exhibited by each child, including directional biases in eye movements, pathological nystagmus, visual field asymmetries, and contrast sensitivity deficits. Our findings indicate that these methodologies will enable the rapid, objective classification and grading of visual impairments in children with CVI, including non-verbal children who are currently precluded from most vision assessments. This would provide a much-needed differential diagnostic and prognostic tool for CVI and other impairments of the visual system, both ocular and cerebral.

## Introduction

Visual impairments can have far-reaching implications for performance in numerous domains of perception and action, but many of the most prevalent disorders often elude clear diagnosis or quantification—particularly disorders that disproportionately affect children. Cerebral/cortical visual impairment (CVI) is the most common source of visual impairment in children in developed countries, affecting 30–40% of children with visual disorders (Hatton et al., 2007; Pehere et al., 2018) and more than 10% of children with any developmental disability (Nielsen et al., 2007). It is predominantly the result of perinatal brain injury, such as hypoxia, a genetic disorder, or head trauma (Roman-Lantzy, 2007), but genetic causes have also been identified (Bosch et al., 2016). Though there is not a consensus on the constellation of symptoms that define CVI (Lueck, 2010; Sakki et al., 2018), it is clear that its presentation is diverse, and it is often diagnosed by exclusion after ocular and geniculate sources of impairment are partially or completely ruled out (McConnell et al., 2021).

A refined and quantitative characterization of any given case of CVI can be highly difficult to obtain, as co-morbid communicative and cognitive impairments frequently preclude children with brain injury from participating in standard vision tests (Good et al., 1994; Huo et al., 1999). Whereas neuroimaging techniques hold promise for distinguishing between some manifestations of CVI (Merabet et al., 2016), behavioral assessment methods that require verbal feedback, comprehension of instructions, or sustained periods of attention are not possible for many CVI patients; even children with intact cognition often find conventional tasks too arduous to complete (Witton et al., 2017). Non-verbal alternatives to common tests do exist, such as visual evoked potentials (Leat et al., 2009; Odom et al., 2016) and preferential looking paradigms (Teller et al., 1986), but these methods are less sensitive than verbal tasks (de Faria et al., 1998) and are suitable for only a few dimensions of visual impairment.

Vision tests based on the analysis of gaze hold promise for the improved diagnosis and quantification of CVI (Caplan et al., 2016; Kooiker et al., 2016; Chang and Borchert, 2020, 2021), including more specific impairments such as visual dysfunction or concussion from traumatic brain injury (Samadani et al., 2015; Samadani, 2016; Barker et al., 2017; Armstrong, 2018; Bin Zahid et al., 2020). Brain injury can impair the magnitude and directionality of saccades (Hunfalvay et al., 2019) and limit the perception of motion or the ability to smoothly pursue moving targets in certain directions, even at slower speeds (Suh et al., 2006). These deficits may be accompanied by a pathological nystagmus, which causes involuntary, repetitive motion in one or both eyes (Sarvananthan et al., 2009). Whereas some of these symptoms may be severe and/or frequent enough to measure passively (e.g., a standing nystagmus), any behavioral assessment of higher-order visual function is encumbered by the requirement to infer function through action, such as the analysis of eye position. For example, the assessment of eye movements in response to the presentation of an object (such as a moving finger) is a fundamental component of a clinical vision exam; if a subject can follow a finger, it is inferred that they can see it. The imprecision with which the assessment is made, however, constrains its capacity to grade visual ability. Brain injury can also impair the ability to perceive and/or react swiftly to targets that appear in different parts of the peripheral visual field (Suchoff et al., 2008), but the perimetry tests that are conventionally used to quantify these impairments (Marín-Franch et al., 2018) require prolonged periods of attention and direction that may be impossible for children with brain injury.

Fortunately, the recent deployment of reliable, consumer-based eye trackers permits more rigorous measurement of eye movements, which in turn increases the potential to detect impairments with more sensitivity and reliability than methods requiring judgments by a human observer. Indeed, automated tasks based on eye-tracking can do far more than this: by measuring eye movements that occur in response to more complex stimuli, and using those eye movements adaptively to drive stimulus alterations, these tasks can rapidly measure other dimensions of visual health with no instructions given and no need for verbal feedback. The most useful ocular responses given by patients in these tasks may be *smooth pursuit* eye movements. As pursuits are extremely difficult to produce in the absence of a visible moving stimulus and highly unlikely to match the trajectory of an unseen, unpredictable target, they provide strong evidence of visual perception (Schütz et al., 2011; Spering and Montagnini, 2011; Spering and Carrasco, 2015; Gegenfurtner, 2016). These tasks consequently have extremely low false positive rates; this is a desirable feature for tests in cognitively impaired children, who are likely to require frequent repeated testing to obtain an adequate amount of valid data. Accurate calibration and head stabilization are often problems when using eye trackers to test individuals with cognitive disorders, but these barriers can be at least partially overcome by designing tasks that rely on accurate positional (rather than derivatives of) gaze data as little as possible and using display-mounted trackers on a mobile monitor, respectively.

We constructed a “Visual Ladder” program of computerized tracking-based tasks designed to assess visual functions that are elicited in a clinical visual exam. This computer program was tested in 10 hospitalized children with varying types and degrees of visual impairment, both with and without an independent CVI diagnosis. We measured the spatial dispersion and magnitude of spontaneous saccades and pursuits, visual field symmetry through saccade latency and directness in response to peripheral stimuli, and spatial visual function (i.e., acuity and contrast sensitivity) from the accuracy of tracking responses to moving noise patches. Children were re-tested with a regularity that depended on available flexibility in their in-patient hospital therapy schedule, school schedule, the child’s overall health on a testing day, and their willingness to participate on that day. We then computed summary metrics of saccades, pursuits, visual field, and contrast sensitivity for each child to determine if the tasks would enable us to (a) place all children on shared scales for each metric and (b) identify the nature and severity of specific visual impairments.

## Materials and Methods

### Observers

Fourteen children between the ages of 3 and 18 years were recruited between July 2019 and March 2021 through doctor or staff referral from the in-patient population at Blythedale Children’s Hospital. The only eligibility criteria were the presence of some form of brain injury or other diagnosed visual impairment, the ability of the child to keep their eyes open, and the ability of the eye tracker to reliably detect their gaze. Four children (with severe impairment or strabismus) could not satisfy this final criterion and were excluded after the first attempted testing session. The gender, age, verbal/non-verbal status, relevant medical history, and clinical vision diagnoses of each child are shown in Table 1, along with the length of time over which they were tested and the total number of Visual Ladder sessions attempted. The total span of testing time depended on the length of the child’s stay in the hospital and the number of sessions varied with the length of their stay, their condition, and their availability. For simplicity, the children are hereafter referred to from C1 to C10, in an order determined by an approximate *post hoc* classification of increasing overall impairment to aid presentation of their results. Blythedale accepts patients below the age of 21, and although the participant recruited at age 18 (C6) and the participant who turned 18 during the study (C5) were not technically “children,” we hereafter refer to all participants as children for simplicity.

**TABLE 1:** Participant information.

Child	Gender	Age (years)	Communicative	Relevant medical history and clinical diagnoses	Testing time	Sessions
C1	M	12	Yes	Large left cerebral ischemic stroke, smaller infarcts in right cerebellum 20/20 vision, previous diplopia	2 months	36
C2	M	12	Yes	Brain tumor (posterior medulloblastoma) 20/30 vision, horizontal nystagmus	4 months	46
C3	F	11	Yes	Complex congenital heart disease, hypoxic ischemic encephalopathy Slow horizontal eye movements; unable to assess acuity	5 months	74
C4	M	16	Yes	TBI, subdural hematomas, encephalopathy from influenza CVI; originally unable to assess acuity, then assessed as 20/25	2 months	18
C5	M	17	No/Yes*	TBI, left subdural hematoma, hypoxia from cardiac arrest Originally unable to fixate/follow and unable to assess acuity, then assessed as 20/25 at end of study	9 months	145
C6	M	18	Yes	Visual impairment from retinitis pigmentosa. Legally blind; possible light perception	6 months	121
C7	F	10	No	Acute cardiac arrest, perinatal hypoxia, optic atrophy CVI; no light perception, no fixate/follow	1 month	15
C8	M	13	No	Perinatal hypoxia, cerebral palsy, Lennox-Gastaut syndrome CVI; no fixate/follow	3 weeks	17
C9	M	5	No	Pelizaeus-Merzbacher disease, neurodevelopmental regression, optic atrophy Upward nystagmus; can fixate/follow with right eye; left eye slow/delayed	2 months	30
C10	F	3	No	Severe global anoxic brain injury, hypoxic ischemic encephalopathy, optic atrophy CVI; no light response	2 weeks	8

*Child C5 was non-verbal for approximately 2 months after enrolling, then regained communicative ability for the remainder of the study.

Parents/legal guardians of each child gave signed informed consent under an approved Institutional Review Board protocol managed by the Biomedical Research Alliance of New York (BRANY). All able communicative children (determined by doctor), gave verbal assent, signed assent, or signed consent, depending on their age and ability prior to being enrolled in the study. Experimental data were secured and managed with the REDCap database (Harris et al., 2009).

### Apparatus

A 27-inch widescreen LCD Dell Optiplex 7760 all-in-one computer running Windows 10 was attached to a mobile trolley using a customized articulated arm. The display was equipped with a Tobii 4C eye tracker (50–95 cm operating distance; 90 Hz sampling rate) with a professional-level license (Tobii Technology, Stockholm, Sweden). Eye tracker data were accessed with the Tobii Pro SDK library, which reports the gaze point on the display for each individual eye and the coordinates of each eye in real space. The raw gaze point data were smoothed with a custom denoising algorithm that avoids smoothing over saccade eye movements. An estimate of mean valid gaze was then computed on each frame by taking the average of both eyes, if both eyes’ data streams were valid on that frame, or just one eye if only one eye’s data stream was valid on that frame. Stimulus behavior was programmed in Python using the Shady graphics toolbox (Hill et al., 2019), which was also used to calibrate screen gamma, and audio feedback was controlled with the Audiomath toolbox (Hill et al., 2021). Minimum and maximum screen luminance values of 10.0 and 221.1 cd/m^2^, respectively, were measured under controlled room illumination with an ILT1700 radiometer (International Light Technologies, Peabody, MA). Observers were measured at a distance as close to 620 mm as possible and our software blanked out the screen and displayed a warning message (which suspended data acquisition) whenever the observer’s eyes were closer than 520 mm or further than 720 mm from the screen. At 620 mm, the display subtended horizontal and vertical visual angles of 51.5 and 30.4 degrees of visual arc, respectively. No other form of distance enforcement or head restraint was used, as this was impractical in the hospital setting. A portable battery was used to power the computer while it was moved around the hospital before being connected to an AC outlet for each test.

### Stimuli

The Visual Ladder program comprised five tasks:

• **Bubble Burst:** multiple colorful bubbles drift around the screen and pop when fixated upon, which prompts the child to generate a multitude of saccades for assessment;
• **Moving Bubbles:** large bubbles appear one at a time and move along preset paths, which assesses smooth pursuit tracking ability;
• **Field Bubbles:** small bubbles appear one at a time at predetermined peripheral locations, which uses fixation latency to assess visual field sensitivity;
• Our **Gradiate** task, which infers the user’s contrast sensitivity function (CSF) by having them track noise patches of varying contrast and noise scale around the screen (Mooney et al., 2020); and
• A **full-screen variant of Gradiate**, in which the same noise patterns cover the screen and move only horizontally, for observers who have difficulty tracking smaller targets.

**FIGURE 1:**
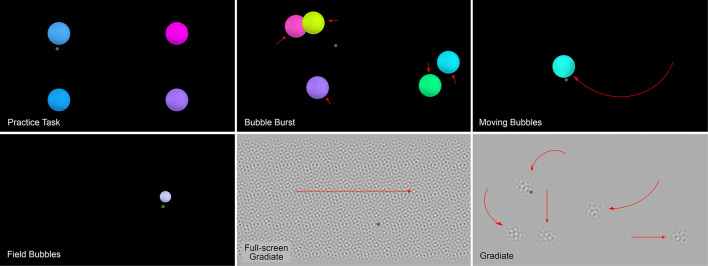
Screenshots of the practice task **(top-left)** and the five Visual Ladder tasks. The green dots and red arrows represent example gaze points and stimulus motion vectors, respectively, and were not visible to the observer during the task.

These five tasks ran automatically in sequence (following a brief practice task) after the program was launched by the experimenter. The sequence is as specified above, except the full-screen version of Gradiate was run before the standard version to place the most difficult task at the end of the sequence. Figure 1 depicts a screenshot from the practice task and from each of the five experimental tasks. The tasks were designed to appear as “games” and combined real-time visual stimulus manipulation, eye tracker input (including denoising procedures and eye movement classifiers), and engaging audio feedback in the form of music and interactive sound effects. A random music track from a collection of songs was played in the background of each task. The goal was to design positive testing experiences that could feasibly be conducted repeatedly for children, rather than an overly sterile, scripted, or non-interactive experience that could theoretically produce more accurate data but, in practice, fail to motivate participation and hence produce little or no results. Another key to sustained motivation in children is brevity (Witton et al., 2017). Our tasks were designed to act upon gaze information and detected eye movements as rapidly as possible, frequently modifying stimuli in the very next display frame in response, and task time limits and trial counts were refined through pilot testing to avoid fatigue while collecting as much useful data as possible. A detailed description of each task is given below.

In the **practice task**, four randomly colored circular “bubbles” with radius 3° each appeared in the center of one of the display’s four quadrants. When the participant’s mean gaze fell within 3.6° of the bubble’s center (its radius plus a twenty percent buffer) and remained within that radius for a total of 1 s, the bubble “popped” with a pitch-randomized pop sound effect and visible bursting animation. The bubble vibrated with increasing amplitude as it approached this popping time, but this vibration was gradually dampened back toward zero—and progress toward the bubble’s 1-s popping timer was gradually lost—if the participant’s gaze left the detection radius. Each bubble’s color was randomly chosen in hue/saturation/value (HSV) color space by combining a random hue between 0 and 1, a random saturation between 0.5 and 1, and a maximally bright value of 1. Each bubble also had a 10% chance of receiving a color saturation of 0 instead (white). Similar popping bubble stimuli were also used in the first three tasks of the Ladder. When all four bubbles were popped, the first task (Bubble Burst) began.

The **Bubble Burst** task was designed to elicit a relatively unbiased distribution of spontaneous saccades. Five bubbles with a diameter of 6° appeared at random locations on the display and drifted randomly around it at a speed of 4° per second in smooth, curving arcs, overlapping when necessary. A random bright color was chosen per bubble in the same way as the practice task. Whenever a bubble collided with an invisible boundary that excluded the outer 20% of the screen’s width and height, it bounced off and moved in the opposite direction. Unlike the vibration-based popping system used in the practice task, each bubble in Bubble Burst was given a random number of “health points” between 10 and 60 and lost one health point per frame in which the observer’s mean gaze point was within the 3.6° detection radius of that bubble, audibly popping when its health reached zero. This health-based system was used so that bubbles were easier to pop overall, as the task was designed to encourage saccades rather than assess fixations. Whenever a bubble was popped, a new randomized bubble appeared elsewhere on the display simultaneously to replace it, ensuring that five bubbles were always visible at once. The task ended after a total of 40 bubbles had been popped.

The **Field Bubbles** task was designed to measure response latency to stimuli appearing abruptly at 48 preset locations in the observer’s peripheral visual field, similar to a perimetry test (Marín-Franch et al., 2018). These locations appeared at twelve 30° angular intervals along four ellipses whose semi-minor and semi-major axes were both set to 15, 20, 25, and 30% of the display’s width and height, respectively. These axes corresponded to 8°-17° eccentricities horizontally and 4°-10° eccentricities vertically.

In each trial, a smaller white bubble with a radius of 1.5° was presented on a black background at one of these 48 locations relative to the observer’s current gaze point. Latency was computed as the time from the bubble’s appearance until the observer’s gaze was within 5° of the bubble’s center. The bubble turned slightly green when detected. The task then waited until the observer popped the bubble using the same vibration system as the practice task, but only 0.5 s of continuous gaze within the detection radius was required to pop it. The next trial began as soon as the bubble popped, using the observer’s actual current gaze point (rather than the last bubble’s position) as its origin. If the observer did not reach the detection zone within 5 s of the bubble’s appearance, the bubble disappeared, and a null result was recorded at that location. In this case, the next trial began from the observer’s current gaze point.

A randomized queue of all 48 field locations was generated at the start of the task. For each trial, the task iterated over the remaining untested locations and selected the first location that could be presented given the observer’s current gaze position (the location of the previous trial’s target). If none of the remaining locations could be tested, a dummy “setup” trial was generated at a location no closer than 5° that ensured that the first remaining location in the queue could be tested next. The first trial was also a dummy trial at a random location at least 5° away from the observer’s current gaze point.

The **Moving Bubbles** task was designed to encourage and assess smooth pursuit eye movements. Ten bubbles with a diameter of 6° appeared one at a time and moved randomly around the screen. Bubble color was again randomly chosen in the same way as the practice task, but here, the bubbles followed random paths along a preset grid instead of the random steering motion used in that task. Each bubble started with 20 “health points” before popping, but unlike in Bubble Burst, a health point was only subtracted on each frame in which the observer had been smoothly tracking the bubble’s trajectory for at least five consecutive frames. This smooth trajectory match was detected using the same algorithm as Gradiate (Mooney et al., 2020), which is described further below. When the bubble had no health points remaining, it popped, and was replaced by a new bubble at a random location at least 10° away on the display. The task ended when ten bubbles were popped this way. To prevent the task from continuing indefinitely for children who could not pursue the bubbles, each bubble also disappeared 8 s after appearing, and the task itself ended prematurely after five bubbles disappeared this way.

The **Gradiate** task for measuring the CSF was validated previously in healthy adults and children (Mooney et al., 2020). In the task, five windowed circular patches of filtered spatial noise with radii subtending 3° followed random, smooth, non-colliding trajectories at a speed of 5° per second on a mid-gray background. At any given time, the noise pattern in each patch was defined by a particular combination of spatial frequency and contrast, which corresponded to a point in 2D logarithmic CSF space. All five targets began with a spatial frequency of 1 cycle per degree (cpd) and a root-mean-square (RMS) contrast ratio of 0.2, but their appearance progressed along different radial “sweep” vectors in CSF space whenever they were tracked by the observer (a behavior that strongly implies seeing). Tracking was detected using a hybrid algorithm, which requires the observer to exhibit a positional match (i.e., the observer’s gaze must be close to the target) and either (a) a smooth trajectory match (i.e., the observer’s recent gaze path must match the recent path of the target, modulo current position) or (b) a saccadic match (i.e., the observer must exhibit frequent catch-up saccades toward the target). The saccadic tracking option ensures that observers who have difficulty with smooth tracking, or who can only smoothly track for short bursts, are still able to generate valid evidence of seeing. After sufficient evidence of tracking was collected this way, the stimulus abruptly shifted to its next point along the radial sweep by changing its spatial frequency and (with the exception of one sweep that moved horizontally through CSF space) contrast. Tracking progress was reset for each new step in each sweep to prevent false positives caused by lingering tracking behavior from the previous step. Progress along any sweep was also accompanied by a glockenspiel sound effect to provide positive feedback to the observer. When the observer allowed enough time to pass without tracking any of the five presented targets, five spatial vision thresholds were inferred simultaneously from the targets’ final appearance, which can be interpolated to obtain an estimate of the complete CSF. In each session of the Visual Ladder, we measured two repeats of a five-point CSF using this standard version of Gradiate, i.e., two trials containing five moving stimuli each. After the task was completed, a screen appeared telling the observer how many musical notes they had generated during the task, which was highly motivating for several children across their numerous sessions with the Visual Ladder.

We also created a **full-screen variant of Gradiate** that replaces the circular noise patches with a full-screen noise pattern scrolling horizontally at 5° per second. The goal of this task was to measure CSFs in highly impaired children who may find the standard version of Gradiate too difficult for any reason (e.g., difficulty making vertical smooth pursuits, attentional deficits, sensitivity to stimulus crowding). In principle, the full-screen version of Gradiate needs only to elicit a low-level optokinetic nystagmus response (OKN) instead of the more precise, curving smooth pursuits required for progress in standard Gradiate; the same approach has been validated previously in healthy subjects (Dakin and Turnbull, 2016). This variant effectively reduces false negatives—children who can see the stimulus, but are unable to track standard Gradiate patches—at the cost of increasing false positives, as there is no positional component to tracking and it is easier for observers to intentionally or unintentionally (e.g., due to incidental nystagmus) match the velocity of the scrolling pattern. Only one CSF sweep could be measured at a time, but the task otherwise behaved in the same way as standard Gradiate, with stimuli progressing along the same five sweeps in CSF space and a glockenspiel sound played after each successfully tracked sweep step.

To further accommodate impaired children, two additional tools were granted to the experimenter during full-screen Gradiate:

• They had the ability to set the direction of the drifting noise (leftward or rightward) for each child, based on qualitative evidence of an inability to track in one direction. The direction of motion was otherwise chosen randomly for each trial.
• They had the ability to switch the noise pattern with a detailed landscape of cartoon characters scrolling with the same speed and direction. This feature provided a way to practice smooth movement on a larger, more visually pleasing cartoon image than the higher spatial frequency noise stimulus, and to recapture the attention of children who were no longer looking at the screen, or otherwise failing to attend to the task, before switching back to the noise stimulus (a “bait and switch” approach). To prevent lingering tracking of the cartoon from causing false positives, all tracking behavior was disregarded for 2 s after switching back to the noise stimulus.

Only one repeat of a five-point CSF was measured with full-screen Gradiate. As in standard Gradiate, a feedback screen informing the observer of how many musical notes they had produced was shown after the task.

### Procedure

Children were tested in their rooms at Blythedale Children’s Hospital. Some sat up in their wheelchairs or in their bed to participate and others were accommodated while lying down according to their physical needs. The display was positioned in front of the child, approximately 620 mm from the child’s eyes, orthogonal to their head pose and line of sight, using the articulated arm that was mounted on the mobile trolley. Children were asked to keep still during the procedure to maintain this distance; for all children, the experimenter attempted to account for unexpected head or body movements by moving the display as necessary. The time of day for testing varied both between and within children due to their hospital schedules. Room illumination was not controlled or measured in the hospital, as children were tested in different rooms surrounded by other equipment (often shared with other patients), but direct sunlight was avoided, and curtains were drawn when possible. We have previously shown that variation in artificial room illumination does not significantly impact the results of our contrast sensitivity assessments (Mooney et al., 2018). Children were always awake and fed well before testing and were thus adapted to the photopic conditions of the experiment.

Each Visual Ladder session was preceded by a one-point calibration step and a simple practice task to confirm the eye tracker was detecting gaze. In the calibration step, a white gear subtending 5° appeared in the center of the display on a black background. When the participant’s mean gaze point (the average of their left and right eyes’ gaze points if both were valid, or just the valid eye) was within 5° of the gear’s center, the program assumed that the participant was looking at the wheel and adjusted an internal calibration variable to account for the difference. The gear spun with increasing frequency while the mean gaze point was within this detection radius and slowed while it was not. The calibration step ended, and the gear disappeared, when the gear’s rotational velocity reached 1080°/s (approximately 2 s of continuous calibration). This calibration step sometimes took up to several minutes, and gaze was no doubt miscalibrated in multiple sessions (particularly for the more impaired children), but our tasks were designed to be resistant to minor-to-moderate calibration errors. The Field Bubbles task is most reliant on calibration, but participants who could not complete the calibration step were likely to perform very poorly on this task in any case and miscalibration is unlikely to introduce specific directional biases in performance.

While the Ladder proceeded automatically and uninterrupted in most testing sessions, the experimenter had a wireless keyboard with several pragmatic controls available to handle the unpredictable barriers and time constraints that often arose while testing:

• They could toggle a trio of small green gaze marker dots, representing left, right, and mean gaze point on the display, to debug cases where the eye tracker could not detect the child (e.g., due to strabismus or unusual difficulty positioning the screen). These markers were enabled to check the status of the eye tracker, typically during the practice task, and were disabled as soon as the experimenter was satisfied with the setup.
• They could check the gaze distance to make sure the child was close to 620 mm, typically during calibration. Distance was checked as needed throughout the tasks if the child could not remain still for the whole session.
• They could end a task prematurely and skip to the beginning of the next task. Each child’s available testing time was sometimes as short as 10 min, and while the Ladder can be completed in that time by a child with only mild or moderate impairment, some tasks (e.g., Field Bubbles) can take significantly longer if every trial is allowed to time out (e.g., due to severe inattention). The ability to skip tasks was added to ensure that impaired children with shorter time slots were able to try later tasks, such as full-screen Gradiate, without having to wait through a long sequence of failures in earlier tasks such as Field Bubbles. Conversely, it was sometimes used to skip full-screen Gradiate in favor of standard Gradiate for children who had previously demonstrated an ability to participate in the latter task but had tight schedules. The experimenter also had the ability to skip the calibration step and practice task for children who could not direct their gaze to specific targets (but who may nevertheless generate useful saccade and pursuit data throughout the program).
• They could toggle the background music on or off at any time. Music appeared in most cases to improve participation and motivate the children, but also appeared in some instances to be a distraction.

As is commonplace for clinical sessions with children, the experimenter freely encouraged them to engage with the procedure and praised their performance, regardless of whether the child was communicative, and interacted freely with communicative children. Rarely, the full testing session was ended prematurely due to the child’s time constraints or a medical or behavioral issue that precluded further participation. No cases of severe adverse health effects were attributed to the procedure.

### Data Analysis

We identified statistical measures for each task in our Visual Ladder program that can be used to compare observers and identify irregularities without being overly sensitivity to the variable number of times each observer was measured or the variable amount of time each observer took to complete a given session. When analyzing saccades and pursuits, for example, we collapsed eye movements into eight directional bins to ensure that interpretable distributions and means could still be generated by the observers who tended to exhibit fewer eye movements, which could be due to intrinsic behavioral factors or eye tracker noise. The results for one child (C5, who was tested over 9 months) were also treated as a longitudinal case study examining recovery from TBI. To contain the scope of this study, all other metrics were computed over the entire duration of each child’s testing period. The primary goal of the study was to establish the ability of the Visual Ladder to produce interpretable results across a heterogenous sample of ten children with diverse medical histories (effectively ten case studies). Statistical tests were hence only used (per participant) to detect broad asymmetries in saccade amplitude—specifically, independent *t*-tests. Our focus was instead on (a) the presence of valid metrics, particularly in non-verbal children, that could be used to aid diagnosis and quantification of visual impairment, and (b) the detection of patterns that are characteristic of ocular or cerebral deficits in the clinical literature.

## Results and Interpretation

### Bubble Burst (Saccades)

**FIGURE 2:**
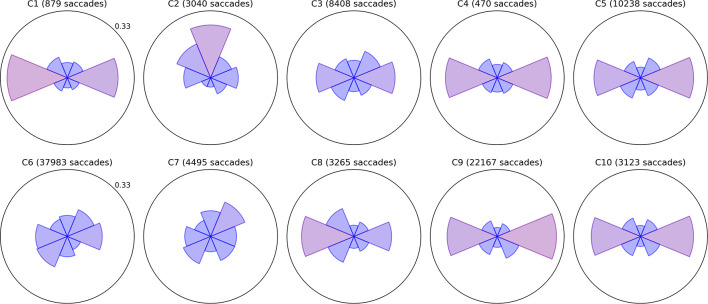
Radial histograms of saccade frequency. Saccades in the Bubble Burst task were binned into eight directions. The upper radial axis limit of each plot is a proportional score of 0.33. The sector bins gradually change in color from blue to red as the proportion for that bin exceeds the mean of 0.125, which allows the more biased directions to be identified at a glance. The participant number and total saccade count are given above each plot.

**FIGURE 3:**
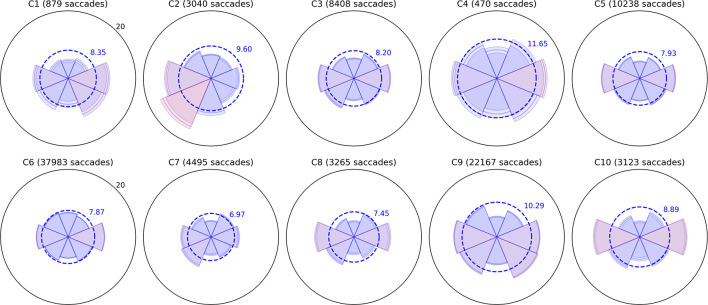
Radial plots of mean saccade amplitude. As in Figure 2, saccades in the Bubble Burst task were binned into eight directions. The radius of each sector represents the mean amplitude of all saccades falling in that directional bin. The overall mean amplitude for each child in degrees of visual arc is denoted by the dotted blue ring, and the color of each sector changes gradually from blue to red as the mean amplitude for that direction exceeds the overall mean. The lighter ring segments at the end of each sector represent the standard error of mean amplitude in that direction.

Bubble Burst is a relatively unconstrained task that aims to bias saccade distributions as little as possible beyond the innate influence of the display’s size and aspect ratio. Bubbles continually appear at random locations as existing bubbles are popped and drift in random directions. We sorted saccades into eight directional bins by computing the angle between the first and last gaze point samples within each saccade, then examined (a) the relative proportion of saccades of any amplitude in each direction and (b) the mean amplitude of saccades in each of those eight directions. Polar plots of these two metrics are depicted for all children in Figures 2, 3, respectively, with values further above each child’s overall mean colored increasingly red for visual clarity. Corresponding panels in each figure represent data from the same child; the same is true for all subsequent two-by-five data figures below. The plots permit spatial abnormalities in the distribution of saccades to be identified at a glance, and provide a numerical quantification of saccade behavior that has the potential to both specify a deficit and enable comparisons between subjects.

Several children exhibit patterns that resemble healthy behavior established by the clinical literature: namely, a bias toward more frequent and larger horizontal saccades than vertical, particularly due to the orientation of the widescreen display (Foulsham et al., 2008), and a weaker bias toward larger (though not more frequent) downward saccades than upward (Collewijn et al., 1988). Visually, these biases produce a familiar “bow-tie” histogram in Figure 2 and (due to the additional downward bias) a “butterfly” distribution in Figure 3. Observers C1, C4, C5, C9, and C10 exhibit both of these patterns with varying degrees of symmetry. C4 and C9 also appear to exhibit larger saccades, on average, than the other participants. Observer C3 exhibits near-normative saccades, but with additional weighting in the top-right and bottom-left directions. On closer examination of recorded video, this pattern appears to be due to a tendency for this child to tilt her head to the left relative to the screen regardless of the experimenter’s attempts to rotate the screen or ask the child to straighten her posture.

A common symptom of various visual impairments, including those caused by brain injury, is a left vs. right asymmetry in ocular and/or attentive behavior. We used independent *t*-tests to confirm several of the horizontal asymmetries in mean saccade amplitude apparent in Figure 3 after categorizing every saccade as either leftward or rightward. Each *t*-test compared the mean distance of all leftward saccades to the mean distance of all rightward saccades for a single participant (i.e., hundreds or thousands of samples per test). The tests revealed significant rightward biases in amplitude for C1 (*t* = 3.155,*p* = 0.002), C6 (*t* = 6.662,*p* < 0.001), and C9 (*t* = 2.031,*p* = 0.042), and leftward biases for C2 (*t* = −11.392,*p* < 0.001), C5 (*t* = −2.807,*p* = 0.005), and C7 (*t* = −3.667,*p* < 0.001). Some of these differences are subtle (e.g., C5 and C7) and would likely go undetected without the use of an eye tracker and repeated testing sessions.

Observers C2, C6, C7, and C8 exhibited a variety of other noteworthy patterns in their saccades. C8 had no significant left vs. right asymmetry in mean saccade amplitude, but his histogram indicates that he saccaded more frequently to the left. Observers C6 and C7 exhibited directional histograms that are unusually isotropic, which suggests that the display may not have been visible to them or not well attended. Indeed, C6 is legally blind, and while he verbally reported being able to see some bubble stimuli, his saccades are unlikely to conform to the environment-driven patterns typical in seeing observers. C7 is a non-verbal child with a CVI diagnosis, and her isotropic saccade histogram suggests that she may have only had minimal awareness of the display or task; this corroborates ophthalmological reports indicating that she has no light perception or fixate-and-follow response (Table 1). Finally, observer C2 exhibited a saccade histogram that was strongly biased upward, indicating a pathological oculomotor behavior that will be discussed further below.

### Moving Bubbles (Pursuits)

**FIGURE 4:**
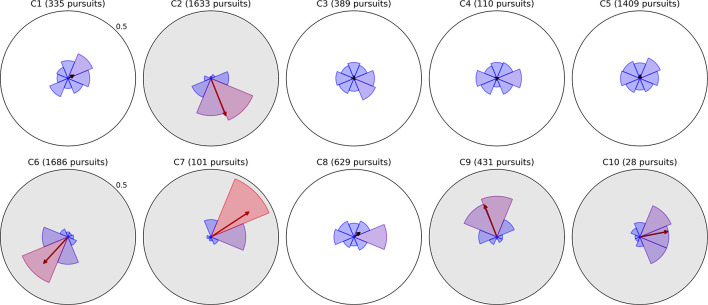
Radial histograms of pursuit frequency and mean bias vectors in Moving Bubbles. Pursuits in the Moving Bubbles task were binned into eight directions. The upper radial axis limit of each plot is a proportional score of 0.5. As in previous figures, the sector bins gradually change in color from blue to red as the proportion for that bin exceeds the mean of 0.125. The participant number and total pursuit count are given above each plot. The arrow originating from the center of each plot represents the Cartesian mean of all pursuits’ unit direction vectors, and thus indicates the direction and strength of an overall bias in pursuit direction. The five plots with gray backgrounds exhibit a particularly strong directional bias and have corresponding radial histograms from Field Bubbles shown in Figure 6.

**FIGURE 5:**
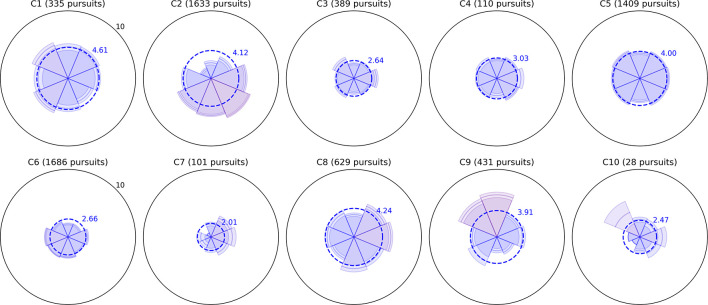
Radial plots of mean pursuit distance computed as path length between the first and last gaze points in the pursuit. As in previous figures, pursuits in the Moving Bubbles task were binned into eight directions and the radius of each sector represents the mean distance of all pursuits falling in that directional bin. The upper radial axis limit of each plot is 10° of visual arc. Overall mean distance is denoted by the dotted blue ring and the color of each sector changes gradually from blue to red as the mean distance for that direction exceeds the overall mean. The lighter ring segments at the end of each sector represent the standard error of mean distance in that direction.

Pursuits were binned into eight directions in the same way as saccades, and corresponding polar plots of directional histograms and mean pursuit distance (computed from pursuit path length) are depicted for all ten children in Figures 4, 5, respectively. The arrow superimposed on each histogram in Figure 4 represents overall pursuit direction bias, computed as the Cartesian mean of the unit direction vectors for all pursuits, and is colored between black and red according to length. As with the saccade plots, these pursuit plots enable a clinician or researcher to quickly distinguish spatial abnormalities in the distribution of pursuits, but also offer more detailed quantification of pursuit ability.

As pursuits guided by the bubble target were equally likely to occur in any direction, we expected the directional distribution and mean distance of pursuits to be relatively isotropic for an unimpaired observer. Observers C1, C3, C4, and C5 fit this pattern, with no directions containing too few or unusually short pursuits, and are hence, less likely to have any severe impairment to their ability to pursue targets. Observer C8 exhibited a small bias toward rightward pursuits in both frequency and distance, but otherwise conformed to a mostly isotropic pattern. Notably, this bias is in the opposite direction to their saccade bias in Figure 2, suggesting that they may have a mild tendency to pursue rightward that requires more frequent corrective leftward saccades.

**FIGURE 6:**
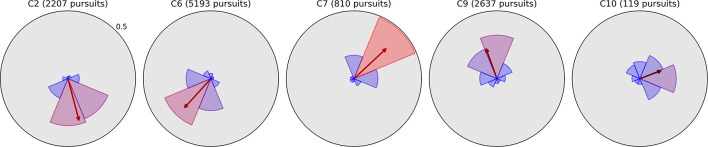
Radial histograms of pursuit frequency and mean bias vectors in Field Bubbles for the same five children whose plots have gray backgrounds in Figure 4 (the same histograms from Moving Bubbles). All elements have the same meaning as in Figure 4 and are essentially identical to that figure for each of the five children despite the absence of moving stimuli Field Bubbles, indicating that their pursuits are likely caused by involuntary nystagmus.

The remaining observers exhibit pursuit asymmetries ranging from large to severe; the directions of these asymmetries are clearly visible in Figure 4, and generally correspond to the directions of greatest mean distance in Figure 5. It is difficult to infer, from Moving Bubbles alone, whether these asymmetries represent a varying ability to track visible targets in certain directions or a pathological nystagmus that is causing smooth eye movements independently of any stimulus. To determine this, we also analyzed pursuit histograms from the Field Bubbles task for observers C2, C6, C7, C9, and C10 (gray backgrounds in Figure 4). Field Bubbles contains no motion at all, and any on-screen pursuits that occur during the task are therefore almost certainly caused by nystagmus. These histograms are shown in Figure 6. They are almost identical to the corresponding histograms for all five children in Figure 4, which suggests that pathological nystagmus is almost entirely responsible for the asymmetries detected in Moving Bubbles rather than an inability to track moving stimuli in certain directions. The mean bias arrows in Figure 6 can consequently be interpreted as a precise quantification of the mean direction and spread of each child’s nystagmus. Notably, observers C2, C6, C7, C9 exhibited significantly larger saccades in the direction approximately opposite to their nystagmus (see previous section), but only observer C2 appeared to exhibit *more frequent* saccades in the opposite direction to his nystagmus. Observer C10, by contrast, did not exhibit any visible signs of a nystagmus in her saccade data. Notably, of these five children, only C2 and C9 had a nystagmus diagnosed in their ophthalmological report, which suggests that nystagmus in C6, C7, and C10 may not have been discernible to a clinical examiner, may have developed more recently than their clinical exam, or may be transient.

Finally, we note that while there are apparent differences in mean pursuit distance across observers in Figure 5, it is difficult to draw inferences from this. Different observers tend to generate different degrees of eye tracker noise, depending on factors such as strabismus, overall movement and attention during the task, the experimenter’s ability to position the display optimally, and the child’s ability to fully open their eyes. As eye tracker noise can interrupt ongoing pursuit detection, e.g., causing one long pursuit to be broken up into several smaller pursuits, it can have a direct impact on mean pursuit distance for each child. We consequently err on the side of caution when interpreting the clinical relevance of a smaller mean pursuit distance, and instead focus on relative differences across directions for each observer. Our pursuit metrics regardless provide an efficient way to quickly visualize overall impairment, precisely quantify specific deficits, and make comparisons between observers.

### Field Bubbles (Visual Field)

**FIGURE 7:**
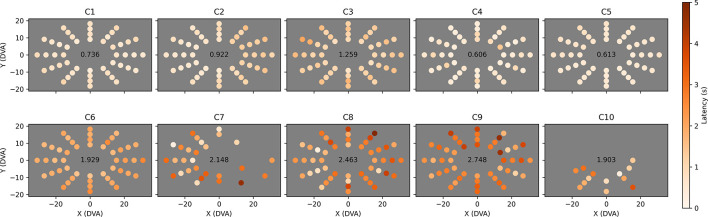
Mean saccade latency across the visual field for each child. Each dot represents the Cartesian coordinates in degrees of visual arc of one of 48 tested locations. The dot’s color indicates mean saccade latency for all valid (i.e., completed) trials to that location, provided that at least three valid trials occurred, on a color map shared by all ten observers ranging from zero (white) to five (red) seconds. Locations at which less than three valid trials were completed are left empty (C7 and C10). Overall mean saccade latency is shown in the center of each plot.

**FIGURE 8:**
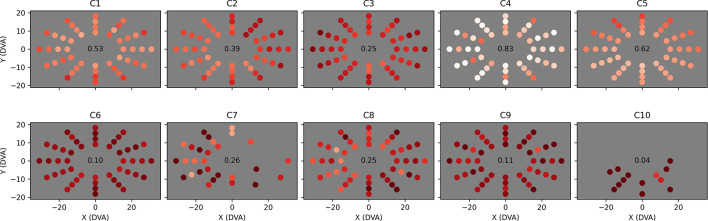
Proportion of saccades to each tested location that were direct. The layout of each plot is identical to Figure 7, but the color of each dot now represents the proportion of valid trials at that location that were completed with direct eye movements only (i.e., eye movements that did not stray out of a narrow strip between the start location and the target location), ranging from dark red (0% direct) to white (100% direct). Locations at which less than three valid trials were completed are left empty (C7 and C10). Overall mean direct proportion is shown in the center of each plot.

We combined all valid Field Bubbles trials (i.e., trials that did not reach the 5-s timeout) at each location for each child and computed mean saccade latency time (Figure 7) and the proportion of trials that were completed without first saccading in an incorrect direction (Figure 8). In Figure 7, a shared color scale is used for the mean latency for each child and target location. Standard error is not shown, but locations with less than three valid trials were excluded from the analysis. The data reveal that observers C1 to C5 found essentially all peripheral targets faster than observers C6 to C10. Observer C6, who is highly communicative but legally blind (i.e., understood the goal of the task and offered extensive feedback, but was expected to have difficulty seeing the stimuli), tended to find the target in a reliably delayed fashion (2–3 s). Observer C7 only reliably generated sufficient data in leftward directions, indicating a potential right-hemifield impairment, and observer C8 was similarly able to find leftward targets faster than rightward targets overall. Their results indicate that visual field deficits may now have the potential to be recognized and quantified in children with brain injury who cannot participate in standard visual field tests. Observer C9 exhibited overall latency impairment but no clear directional bias. Among the less impaired observers, observer C2 reacted more slowly to rightward targets and observer C5 reacted slightly slower to targets in the upper-left field quadrant.

The proportions of trials completed through direct saccades only (Figure 8) are potentially more informative than saccade latency. Whereas all trials are potentially useful in identifying certain directional impairments (e.g., directional oculomotor deficits or broad attentional asymmetries), only trials in which the observer saccaded directly toward the target provide strong evidence of localized visual field neglect or a field cut, as any other eye movements will naturally change the target’s relative retinal location. These “direct saccade” trials were defined as trials in which the observer found the target while keeping their gaze inside a narrow strip (5° in width) extending from their initial fixation point to the target. The observer was not required to saccade to the target in one eye movement, as this would exclude children who can only reach distant targets through a sequence of small saccades. The data in Figure 8 confirm that observers C1 to C5 performed better at Field Bubbles than observers C6 to C10 (with C4 performing particularly well) and displayed the same directional impairments for C2, C5, C7, and C8, but in combination with the mean latencies, contain several other implications:

• C3 made fewer direct saccades than the other children with comparably low mean latency. This could suggest a general deficit in attention or perception that the observer made up for with a motivated follow-up search.
• C6 (age 18, verbal, legally blind) made direct saccades in only 10% of trials, with no clear directional bias in either directness or latency, suggesting that he may have simply saccaded around the display randomly until his gaze collided with the target.
• C9 (age 5, non-verbal) exhibited a similarly low proportion of direct saccades (11%), but had worse latency overall than C6, suggesting that his search for the target may have been less motivated.

The eye tracking-based measures of ocular movements introduced here were designed to quantify the behaviors that are normally used in clinical exams to infer the ability to see in non-communicative subjects. The results show these measures provide the opportunity to grade impairments of visual function in children following brain injury with higher fidelity than is currently practiced.

### Gradiate (Contrast Sensitivity)

**FIGURE 9:**
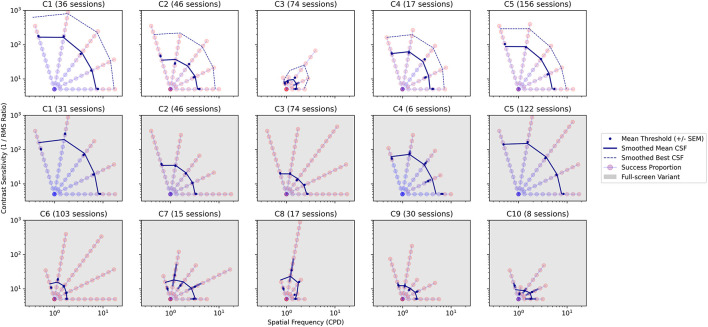
Gradiate CSFs from the standard variant for observers C1 to C5 (top row) and from the full-screen variant for all observers (middle and bottom rows, gray background). The horizontal and vertical axes (both log) represent spatial frequency in cycles per degree and RMS contrast sensitivity, respectively. Each colored circle represents one step in a radial sweep of stimuli progressing outward (and becoming harder to see) from a common origin. The color of each circle varies from red to blue depending on the proportion of presentations of that stimulus in which the observer successfully tracked it (with blue denoting a higher success rate). The solid blue line in each panel is a smoothed CSF interpolated from the mean thresholds of all five sweeps. In the top row, a dotted blue line is also shown representing a smoothed CSF interpolated from the best thresholds of all five sweeps. The total number of sessions for each child/task is shown above each panel.

Observers C1 to C5 were able to track moving stimuli sufficiently well (see Figures 4–6) to enable measurements of contrast sensitivity using our Gradiate task. Multiple Gradiate thresholds were measured using a standard version of the task, with five moving noise patches presented at once for tracking. Observers C6 to C10 were only able to generate thresholds with the full-screen variant. The combined results from both versions of Gradiate are shown in Figure 9. The top row depicts standard Gradiate CSFs for observers C1 to C5; observers C6 to C10 could not complete even a single trial of standard Gradiate across all sessions, which, while obviously indicative of extensive visual or attentional dysfunction, does bolster our previous conclusion that Gradiate is resistant to false positive responses (Mooney et al., 2020). The bottom two rows (gray background) depict full-screen Gradiate CSFs for all ten children. In all panels, each circle represents one combination of spatial frequency and contrast, and the redness of the circle indicates the proportion of trials in which the observer was able to successfully track that stimulus. For standard Gradiate, both mean CSFs (solid blue lines) and best CSFs (dotted blue lines) are shown. As Gradiate is highly resistant to false positives, the best CSFs (dotted lines) are likely to be valid estimates of the observer’s sensitivity under optimal conditions for that child, such as high motivation and rapt attention. For full-screen Gradiate, only the mean CSFs are shown, as this version of the task is more susceptible to false positives and the best scores cannot be safely interpreted as valid. (The irregular lengths of the “best” thresholds in each sweep of this task, compared to the largely correlated lengths in the top row, are strong evidence of some false positives in the full-screen variant.) The number of sessions is shown above each panel. Note that the number of full-screen Gradiate sessions is lower than the number of standard Gradiate sessions for C1, C4, and C5 due to some instances of full-screen Gradiate being skipped by the experimenter to meet the child’s scheduling constraints (given their demonstrated ability to complete standard Gradiate).

As anticipated, children who were able to generate data in standard Gradiate (C1 to C5, top row of Figure 9) generated similar mean CSFs in the full-screen variant (middle row of Figure 9), indicating that the full-screen variant retains Gradiate’s validity. However, as was also expected, some children were able to occasionally generate much higher thresholds in the full-screen variant than their best thresholds in the standard variant—particularly C3 and C5, likely because they completed a much larger number of Gradiate sessions than the other children and thus had more opportunity to generate more exaggerated full-screen thresholds. Unsurprisingly, the children who were only able to generate data in the full-screen version of Gradiate (C6 to C10) tended to exhibit more impaired mean CSFs. Medical histories indicate that these children are legally blind (C6), lacking in light response (C7, C10), or unable to fixate or follow in at least one eye (C8, C9). None were able to have their acuity assessed by the ophthalmologist—nor was C3, who was nevertheless able to produce a weak CSF with standard Gradiate. Four of these children (all except C8) also exhibited pathological nystagmus (Figure 6), which makes tracking-based tasks like Gradiate difficult: the observer will likely find pursuit in arbitrary directions difficult, while also being particularly susceptible to false positives if the velocity of their repetitive nystagmus coincides with the more restricted movement of the full-screen stimulus. As we have discussed in our previous work, it is difficult to make the Gradiate task more accessible to observers with smooth pursuit disorders without increasing the error rate to an unusable extent (Mooney et al., 2018, 2020). The full-screen variant generates higher contrast sensitivity thresholds in general, as the criteria for successful tracking are easier: there is no positional gaze component required and only two directions in which the stimulus can move. It is nevertheless likely that some of the successful tracking exhibited by observers C6 through C10 in full-screen Gradiate is valid—particularly for C10, who tracked the most visible stimuli more reliably (out of her eight sessions) than the other four most severely impaired children. Despite these caveats, we were able to demonstrate that a high-quality measure of spatial vision—among the most informative tests of visual health and impairment—can be obtained in children with brain injury, including those who are impaired in their ability to communicate. Taken together, the results of the Gradiate measures show that all observers, even the child deemed legally blind, have spatial visual function that can be quantified and compared across observers. The use of a full-screen variant of Gradiate is also justified in children who cannot complete the standard five-ball task, since it may have been concluded that such participants had no spatial visual abilities if the full-screen tests were not administered.

### Longitudinal Recovery From TBI

**FIGURE 10:**
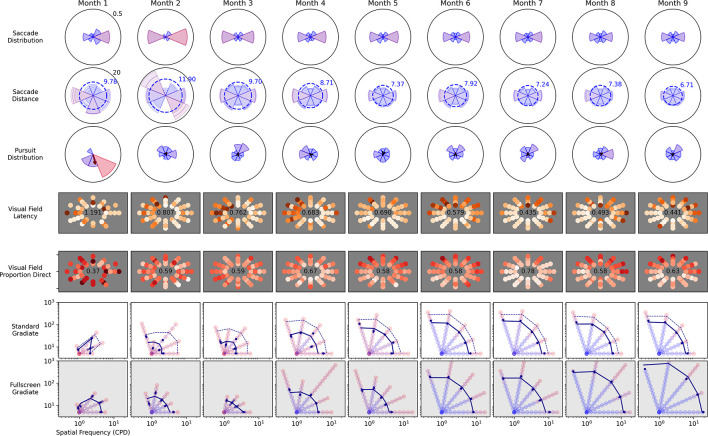
Longitudinal recovery from a traumatic brain injury for observer C5 over 9 months. The child was admitted to Blythedale Children’s Hospital approximately 3 months after accident and immediately enrolled in the study. He was non-communicative for approximately 2 months post enrollment. All plot layouts, axes, and units are identical to their corresponding previous figures.

Observer C5 was measured over the course of 9 months, following a traumatic brain injury, and completed enough sessions of the Visual Ladder to highlight the Ladder’s potential longitudinal use. Figure 10 depicts monthly metrics from many of the analyses presented above. From top row to bottom row, the metrics are saccade directional histograms, mean saccade distance, pursuit directional histograms, visual field latency, visual field direct proportion, standard Gradiate, and full-screen Gradiate.

The data reveal marked improvement across most metrics. Large asymmetries in both saccades and pursuits disappeared by Month 3, and mean saccade latency in Field Bubbles steadily decreased over the first 6 months. Notably, the proportion of direct saccades in Field Bubbles did not noticeably change after Month 2; furthermore, while an early leftward impairment in field latency disappeared around Month 5, an upward/rightward impairment in latency appeared at the same time and persisted through the remaining months. Most remarkably, the observer’s CSF improved consistently over the first 6 months in both the standard and full-screen Gradiate variants, which of all the depicted metrics most directly implies neurological recovery in spatial visual function. The longer recovery time exhibited in the patient’s CSF and mean peripheral saccade latency (∼6 months), compared to the quicker recovery of his basic eye movement distributions (∼2 months), reinforces the importance of higher-level visual function assessment in providing a more comprehensive picture of recovery from brain injury. Our data are also consistent with his ophthalmological reports: C5 was unable to even fixate-and-follow in his examinations on arrival and after 2 months, but at the end of our study (when he was discharged) was able to have his acuity measured as 20/25. The important difference is that the Visual Ladder measurements were better able to grade the changes over time. Overall, his results demonstrate that the approach we have developed enables the ability to measure visual impairments in children after brain injury and quantify recovery longitudinally.

## Discussion

The study results demonstrate that rapid, intuitive assessments based on eye-tracking have promise for aiding the classification and quantification of visual impairment, including diffuse conditions such as CVI. The outcomes of the Visual Ladder can provide intuitive visualizations for clinicians that allow for rapid detection of broad asymmetries, directional biases, and spatial vision deficits, but just as importantly, they allow numerous types of visual disorder (both ocular and cerebral) to be precisely quantified along multiple dimensions of visual ability. Data are generated efficiently, and our ability to frequently retest the children in the study indicate that our attempt to “disguise” vision assessment behind intuitive game-like tasks was largely successful in sustaining interest and engagement. Below, we highlight three broad outcomes of our study that we find particularly noteworthy.

First, we were able to quantify saccades, smooth pursuits, and contrast sensitivity in children of a wide variety of ages and communicative ability, including a non-verbal child aged just 3 years (C10). This suggests that our game-like approach to assessment is appropriate for both younger and older children and holds promise for the generation of age-specific normative scores in the future. Visual field assessment was particularly difficult for non-verbal children, as it requires more attention that the other bubble tasks or full-screen Gradiate, but we nevertheless observed some broad asymmetries (e.g., a right hemifield impairment for C8) across repeated sessions. Our ability to assess these children with the Visual Ladder and place them on common quantitative scales, despite their other cognitive and communicative deficits, is the most promising takeaway of our study. While the Visual Ladder does not assess many of the higher-level symptoms associated with CVI, such as diffuse attentional impairments, color preferences, and issues perceiving crowded or complex scenes, our in-depth analysis of low-level patterns in eye movements and contrast sensitivity could nevertheless aid clinicians in characterizing CVI or ruling out other explanations for impaired visual function. It is also possible that further analysis of our dataset (e.g., through machine-learning) could reveal statistical fingerprints of disorders such as CVI that are not yet evident to us.

Second, while poor performance on one Ladder metric generally coincided with poor performance on others (seen here most clearly in observers C6 to C10), there are also unique patterns of outcomes across the Ladder tasks that distinguish different participants and affirm the need for a diverse range of quantitative visual tests. Observer C2, for example, performed well in Field Bubbles and standard Gradiate despite exhibiting a strong downward nystagmus, suggesting that his visual deficits may be mostly ocular rather than cerebral. Conversely, observer C3 exhibited relatively normal distributions of saccades and pursuits but had worse overall latency in Field Bubbles than the other verbal children and a highly impaired CSF, which likely indicates that she has intact ocular function but poor spatial vision. The data from observer C6—including his null result for both versions of Gradiate—affirm his status as legally blind, despite evidence of highly motivated participation (e.g., his consistent, though delayed, latency in Field Bubbles). These are patterns that are difficult to extract from any single metric and demonstrate that the Visual Ladder is well-placed to parse some of the numerous and diverse symptoms that can characterize distinct types of visual impairment or even distinct cases of CVI. Identifying the commonalities and dissociations between performance across different aspects of related visual functions—an analytic feat that is not possible without tasks that work for non-verbal children—will likely be fundamental for building a quantitative description of CVI based on objective visual criteria.

Third, observer C4—who was diagnosed with CVI 6 months prior to being enrolled in our study, following a traumatic brain injury—exhibited essentially no impairment across any of our metrics. It is possible that his diagnosis of CVI was based solely on higher-level deficits not measurable by the Visual Ladder, but more likely that they underwent a substantial recovery in the intervening 6 months, similar to the recovery we observed in real time for observer C5 (Figure 10). Establishing a regular program of ongoing longitudinal measurement, as we are in the process of doing at Blythedale Children’s Hospital, would ensure that we can follow such recovery in detail and enhance our understanding of how outcomes after traumatic brain injury differ from outcomes of other sources of visual disorders.

Our tasks assess an expansive array of visual health metrics across many sessions, and our analysis here is certainly not exhaustive. There are many potential modifications and additional metrics that could provide further insights into visual impairment. We did not, for example, examine the temporal frequency of saccades or pursuits; such metrics are particularly susceptible to a confounding variation in eye tracker noise, which is typically a larger problem for children with worse impairment overall (due to inattention, difficulty remaining still or maintaining distance, strabismus, etc.). We did not take monocular measurements due to session time constraints and the intolerance or distraction expressed by many children toward eye patching, particularly the non-verbal participants. Separate assessment of each eye is tractable with our approach and would certainly be an important feature to add to our procedure whenever possible in future studies, particularly when measuring visual field health and spatial vision. For clarity, we also divided our analysis of different visual abilities according to the tasks designed specifically to elicit them, but further cross-analysis between tasks (as we did with pursuits in Moving Bubbles and Field Bubbles to identify nystagmus) could reveal more about certain impairments. A more complete picture of overall saccadic ability, for example, can likely be formed from any of our tasks. The full-screen variant of Gradiate could also benefit from further improvements to its false positive rate, such as better classification of tracking behavior that allows stimulus-driven pursuits to be distinguished from incidental nystagmus or random drift. One way to achieve this may be to estimate the direction of any nystagmus from pursuit data in the preceding bubble tasks, as in Figure 6, and have the full-screen noise stimulus only scroll in the two directions orthogonal to that observer’s nystagmus. We plan to investigate many additional avenues of analysis, both in real-time (to improve interactive stimulus behavior) and *post hoc*, as we continue to refine the Visual Ladder and deploy it in a wider clinical population.

It must also be noted that we were not able to measure all recruited children, despite our efforts in making the tasks as broadly deployable as possible (e.g., designing tasks with minimal reliance on perfect calibration). Four children were excluded from the study after initial testing revealed critical shortcomings in the eye tracker’s ability to capture the user’s gaze or eye position. These shortcomings were generally caused by strabismus (even when the tracker was set to measure only one eye), scoliosis that precluded proper positioning of the display and tracker, and/or frequent blinking that did not desist over time. Gaze-based assessment, while highly promising overall, is not a panacea: it replaces certain forms of behavioral feedback that are often impaired by brain injury (speech, manual responses, etc.) with another form of feedback that is usually more intact and functionally relevant for vision (eye movements), but certain types of disorders can still impair eye movements to an extent that makes our approach unfeasible. We believe that more of these children will become reachable as (a) eye tracker hardware improves, (b) our algorithms for handling transient gaps in gaze data improve, and (c) we find informative metrics that appear even in the noisiest eye tracking data, all of which we plan to pursue in future studies.

While repeated visual assessment does not automatically constitute a form of therapy, it is natural to consider ways in which the Visual Ladder could be modified to take on a more therapeutic role. It already has several advantages in this regard: the tasks it comprises are goal-directed, interactive, and entertaining, which are important ingredients for a successful program of behavioral treatment. The various tasks also naturally reward progress. More active saccading and pursuit behavior in the bubble tasks leads to more rapid popping, which our participants visibly enjoyed, and faster task completion times. More explicitly, both versions of Gradiate have an ability-driven scoring system built into them (number of musical notes heard); many verbal participants remembered their Gradiate scores and expressed enthusiasm upon surpassing them in future sessions. Gradiate also directly pushes participants into more difficult perceptual territory as they continue to make progress. If the bubble tasks were reconfigured to similarly spend more time at the boundaries of each participant’s ability—such as gradually increasing the number of simultaneous bubbles in Bubble Burst, including more trials in high-latency locations during Field Bubbles, or increasing bubble movement speed in Moving Bubbles—they could potentially train participants to improve along the very dimensions of visual function already measured by those same tasks. Anecdotally, hospital staff and patient families have often reported that our repeated visual assessments appear to induce similar levels of stimulation in the children to other therapeutic programs.

The primary goal of the present experiment was to demonstrate that tracking-based assessment can be used to detect, quantify, and compare a variety of fundamental visual abilities, even in non-verbal children with brain injury. Given the numerous and well-known barriers to testing this population, we anticipated a moderate probability of failure, e.g., obtaining mostly null results for most participating children. We consequently did not set out to compare their data to results from an age-matched population of healthy observers. However, having now shown that our approach is promising for impaired children, our priorities for future research are indeed (a) establishing age-matched norms and (b) identifying the metrics that most effectively predict specific patient outcomes, quantify various metrics that are currently (and necessarily) treated as categorical in non-verbal children, and inform choices of actionable therapy. To accomplish these aims, our future studies will involve a larger sample of participants with varying visual disorders, more detailed comparisons of Visual Ladder outcomes with medical history and prognosis, and ongoing collaborations with ophthalmologists and therapists, all with the ultimate goal of refining the diagnosis of diffuse visual impairments such as CVI.

## Data Availability Statement

The raw data supporting the conclusions of this article will be made available by the authors, without undue reservation.

## Ethics Statement

The studies involving human participants were reviewed and approved by the Biomedical Research Alliance of New York. Written or verbal assent and/or consent to participate in this study was given by the participants and/or their legal guardians, depending upon each participant’s age and ability.

## Author Contributions

SM designed and programmed the experiment, analyzed data, created figures, and wrote the manuscript. NA designed the experiment, recruited the participants, collected the data, and edited the manuscript. GP designed the experiment and edited the manuscript. All authors contributed to the article and approved the submitted version.

## Conflict of Interest

SM and GP were co-inventors on pending provisional US patent application 62/749,360, which includes the described Gradiate software, pending provisional US patent application 63/167,220, which includes the described trackability of eye movements and pending provisional US patent application 16/661,596 and Patent Cooperation Treaty Application Serial No. PCT/US19/57638, which includes the described methods for evaluating contrast sensitivity and other eye gaze metrics. The remaining author declares that the research was conducted in the absence of any commercial or financial relationships that could be construed as a potential conflict of interest.

## Publisher’s Note

All claims expressed in this article are solely those of the authors and do not necessarily represent those of their affiliated organizations, or those of the publisher, the editors and the reviewers. Any product that may be evaluated in this article, or claim that may be made by its manufacturer, is not guaranteed or endorsed by the publisher.

## Supplementary Material

Supplementary Video 1 Video demonstrating a segment of all tasks in the order they were presented to participants: Practice Task, Bubble Burst, Moving Bubbles, Field Bubbles, Full-screen Gradiate, and Gradiate. The green marker represents the mean gaze point of the participant (here, an author).
